# Description of the Framingham Heart Study data for Genetic Analysis Workshop 13

**DOI:** 10.1186/1471-2156-4-S1-S2

**Published:** 2003-12-31

**Authors:** L Adrienne Cupples, Qiong Yang, Serkalem Demissie, Donna Copenhafer, Daniel Levy, for the Framingham Heart Study Investigators

**Affiliations:** 1 Department of Biostatistics, Boston University School of Public Health, Boston, Massachusetts, USA; 2 Department of Neurology, Boston University School of Medicine, Boston, Massachusetts, USA; 3 Framingham Heart Study, National Heart, Lung, and Blood Institute, Bethesda, Maryland, USA

The data for Problem 1 come from the Framingham Heart Study, centered in Framingham, Massachusetts. Originating in 1948, the Framingham Heart Study is an ongoing prospective study of risk factors for cardiovascular disease. Two cohorts have been recruited into the Study and a third cohort is currently being recruited. The Genetic Analysis Workshop 13 data set consists of genotyping information from a 10-cM genome scan along with phenotypic information on a number of phenotypes collected from the first 21 examinations of the original cohort and the first 5 examinations of the offspring cohort.

In 1948, the Framingham Heart Study-under the direction of the National Heart Institute (now known as the National Heart, Lung, and Blood Institute; NHLBI)- embarked on an ambitious project in health research. At the time, little was known about the underlying causes of heart disease and stroke, but the death rates for cardiovascular disease (CVD) had been increasing steadily since the beginning of the 20^th ^century and had become an American epidemic. Despite declines in the latter part of the century, cardiovascular disease remains the top cause of death in the US today.

The Framingham Heart Study is now conducted in collaboration with Boston University. Its objective is to identify common factors or characteristics that contribute to CVD by following its development over a long period of time in a large group of participants who had not yet developed overt symptoms of CVD or suffered a heart attack or stroke [1]. The study was one of the first long-term prospective studies and was planned for 20 years initially. It continues today with more than 50 years of follow up on the original cohort.

Framingham, Massachusetts was selected because it was a moderate-sized town with a relatively stable population that was thought to reflect many communities in the US at that time. In the late 1940s it was estimated that about 10,000 individuals were in the age range 30–60 years in this community and thus were eligible for recruitment to a study of approximately 6000 subjects. The researchers recruited 5209 subjects (2336 men and 2873 women) between the ages of 28 and 62 from a systematic 2/3 sample of the households in the town of Framingham, Massachusetts. The plan was to recruit all household members in the age range of 30–60 years within each house that was selected for study. Thus, the sample included 1644 spouse pairs. Although there was no intention to recruit extended families for family studies, many biologically related individuals were recruited, including siblings and some parent-offspring pairs. Since 1948, members of the original cohort have continued to return to the study every 2 years for a detailed medical history, physical examination, and laboratory tests.

The study enrolled a second-generation-5124 of the original participants' adult children and the spouses of these adult children-to participate in similar examinations in 1971. Two thousand six hundred and sixteen of these subjects are offspring of the spouse pairs from the original cohort and 34 are stepchildren. Another 898 offspring are children of cohort members where only one parent was a study participant; they were recruited because their parents exhibited elevated lipid levels. In addition, 1576 offspring cohort participants are spouses of offspring. The offspring cohort has been followed every 4 years (except between Exams 1 and 2 with an intervening 8 years) using protocols similar to those used for study of the original cohort.

In the mid-1990s about 1800 members of the largest 330 pedigrees were selected for a genome scan that was conducted by the Mammalian Genotyping Service in Marshfield, Wisconsin. The expanded pedigrees consist of 4692 subjects, of whom 2885 have participated in the Framingham Heart Study. Among the Framingham participants in these 330 pedigrees, there are 3041 parent-offspring pairs, 2796 sib pairs, 2107 avuncular pairs, 183 grandparent-grandchild pairs, and 1595 first-cousin pairs.

For the Genetics Analysis Workshop 13, we provide data for systolic blood pressure, height, weight, total cholesterol, fasting HDL cholesterol and triglycerides, blood glucose, and appropriate covariates such as age, sex, smoking, and alcohol consumption. These data were provided for Study participants in the 330 pedigrees from the original and the offspring cohorts. Data were provided for the first 40 years of follow up in the original cohort (Exams 1 through 21) and the first 20 years of follow up in the offspring cohort (Exams 1 through 5).

Over the years, careful monitoring of the Framingham Study sample has led to the identification or validation of the major CVD risk factors-high blood pressure, high blood cholesterol, low HDL cholesterol, smoking, obesity, diabetes, and physical inactivity-as well as a great deal of valuable information on the effects of related factors such as blood triglyceride levels, age, gender, and psychosocial factors. Although the Framingham cohorts are primarily White, the importance of the major CVD risk factors identified in this group have been shown in other studies to apply almost universally among racial and ethnic groups, even though the patterns of distribution may vary from group to group [2]. In the past 50 years, the study has produced over 1200 articles in leading medical journals. The concept of CVD risk factors has become an integral part of the modern medical curriculum and has led to the development of effective treatment and preventive strategies in clinical practice.

The Framingham Heart Study continues to make important scientific contributions by enhancing its research capabilities and capitalizing on its inherent resources. New diagnostic technologies, such as echocardiography (an ultrasound examination of the heart), carotid artery ultrasound, and bone densitometry (for monitoring osteoporosis), are evaluated and integrated into ongoing protocols.

While pursuing the Study's established research goals, the NHLBI and the Framingham investigators are expanding their research into other areas such as the role of genetic factors in CVD. One project currently under way is the establishment of immortalized cell lines from surviving original cohort participants and members of the offspring cohort. These cell lines will enable extensive investigation of the genetic etiology of not only CVD, but also other diseases. Framingham investigators also collaborate with leading researchers from around the country and throughout the world on projects in stroke and dementia, osteoporosis and arthritis, nutrition, diabetes, eye diseases, hearing disorders, lung diseases, and genetic patterns of common diseases.

The unflagging commitment of the research participants in the NHLBI Framingham Heart Study has made more than 50 years of research success possible. Further information on the Study can be found at . The Study invites researchers around the world to consider this rare collection of data as a resource that may further their own research. There are established protocols for requesting data and/or DNA samples and investigators are encouraged to pursue research questions using this resource.
